# The Impact of Land Use Transformations on Zooplankton Communities in a Small Mountain River (The Corgo River, Northern Portugal)

**DOI:** 10.3390/ijerph16010020

**Published:** 2018-12-21

**Authors:** Łukasz Sługocki, Robert Czerniawski, Monika Kowalska-Góralska, Magdalena Senze, Anabela Reis, João S. Carrola, Carlos A. Teixeira

**Affiliations:** 1 Department of Hydrobiology and General Zoology, Faculty of Biology, University of Szczecin, 71-712 Szczecin, Poland; lukasz.slugocki@usz.edu.pl; 2 Centre of Molecular Biology and Biotechnology, University of Szczecin, 71-712 Szczecin, Poland; 3 Department of Hydrobiology and Aquaculture, Wrocław University of Environmental and Life Sciences, 51-630 Wrocław, Poland; monika.kowalska-goralska@upwr.edu.pl (M.K.-G.); magdalena.senze@upwr.edu.pl (M.S.); 4 Department of Geology, University of Trás-os-Montes e Alto Douro, 5001-801 Vila Real, Portugal; anarreis@utad.pt; 5 Centre for Mechanical Engineering, Materials and Processes, University of Coimbra, 3000-079 Coimbra, Portugal; 6 Centre for the Research and Technology of Agro-Environmental and Biological Sciences, University of Trás-os-Montes e Alto Douro, 5001-801 Vila Real, Portugal; joao@utad.pt (J.S.C.); cafonso@utad.pt (C.A.T.)

**Keywords:** land use, river bed changes, ecohydrology, organic matter, river continuum

## Abstract

Transformation of the river catchment and the river bed cause significant changes in the functioning of river ecosystems. The main effects of anthropogenic transformations are hydrological changes, such as lower current velocity or an increase of nutrient content, and higher temperature. Zooplankton reacts rapidly to the new environmental conditions in rivers, increasing its richness and abundance. We tried to answer two questions: what type of catchment use has a greater influence on the zooplankton communities in a river and how do dam impoundments influence the zooplankton communities downstream? The study was conducted in the Corgo river (drainage of the Douro river, Northern Portugal) at 17 sampling sites in the lotic, free-flowing sections. Crustaceans present in the Corgo can attain relatively high densities in the rural section, which offers them better trophic conditions. The urban catchment use and the presence of dams have a greater impact on the rotifer density and the increase of zooplankton density downstream. The results of this study confirm that zooplankton properties allow for the evaluation of the degree of river-bed transformation.

## 1. Introduction

Catchment and river-bed transformations cause changes in the river regime, resulting in irreversible alterations of the physicochemical and biological variables [1,2]. Different catchment land uses play a paramount role in the functioning of river ecosystems, and particularly trophic network structures [3]. More extensive agriculture in a catchment area has a significant impact on the organic and inorganic nutrients, especially the nitrogen and phosphorus compounds [4]. It undoubtedly increases the amount of live and dead organic matter in a river, the size of the macrophyte-covered area of river bed, and the abundance of aquatic organisms. 

Anthropogenic changes in the river bed, for example dams or reservoirs, are another factor that positively affects the increase of nutrients in a river. These changes include larger built-up areas and river-bed maintenance practices that secure flood control systems and energy needs, and allow water-retention for agglomeration, crops, and forestry [2,5,6]. The above-mentioned needs are secured by building water-impounding structures that create reservoirs altering the hydrological and biological regime of rivers. Also, long water-retention time and low current velocity in the reservoirs lead to phenomena typical of flow-through lakes and not fast-flowing rivers. Consequently, significant amounts of organic sediments accumulate, aquatic vegetation grows (including phytoplankton and macrophytes), and the river ichthyofauna is replaced by lake ichthyofauna [2,7,8,9]. 

Due to these alterations, typical lake organisms, which usually are not present or expected to be present in a high biomass, can develop and maintain their populations. Zooplankton normally occurs in scarce quantities in small rivers and its abundance is an indicator of such alterations [10,11,12]. However, hydrological changes, such as lower current velocity or an increase of nutrient content, and higher temperatures in small rivers provide atypical yet favorable conditions for zooplankton growth. Zooplankton reacts rapidly to the hydrological changes in rivers, increasing its richness and abundance [13,14]. This certainly depends on the reservoir characteristics, and specifically its hydrological conditions, which consequently can change biological conditions and favor the development of planktonic organisms that cannot resist stronger water currents. Even though zooplankton has been proven to show good indicative properties for evaluating hydrological changes in rivers [15], it has never been used as such an indicator. The authors of the present work attempted to show how zooplankton can be an indicator of hydrological changes in rivers as well as catchment alterations. 

According to the River Continuum Concept (RCC) [3], in large rivers, zooplankton is visible in larger amounts only in the lower section, where the current is the slowest, and stagnant water basins supply the river with zooplankton. It is commonly known that any human-induced alterations to environmental conditions have a transforming effect on the RCC in the downstream sections of a river. The more severe the changes upstream, the stronger the effects downstream [2]. Zooplankton was one of the indicators for these changes [6,14]. However, the functioning of a small lowland river depends on different values of hydrological factors than that of mountain rivers. In lowland rivers, slower current velocity and weaker turbulence can foster a greater reduction in the zooplankton abundance through the sedimentation phenomena and fish predation [13,16]. Moreover, similar to lowland reservoirs, the retention time needed for zooplankton development and maintenance of its great abundance may be too short in mountain reservoirs. Until now, studies showing spatial changes in the zooplankton composition in small mountain rivers in regard to environmental changes in the catchment and river-bed transformations have not been conducted. Besides, zooplankton communities in small and upstream rivers is still poorly documented [17]. 

This study aimed to examine spatial distribution of zooplankton communities in a small mountain river subjected to anthropogenic changes. To achieve the objective, the authors attempted to answer the following questions: (1) What type of catchment use has a greater influence on the crustacean and Rotifera communities in a river? (2) How do dam impoundments influence the zooplankton communities downstream?

## 2. Materials and Methods 

### 2.1. Study Area

**Figure 1:**
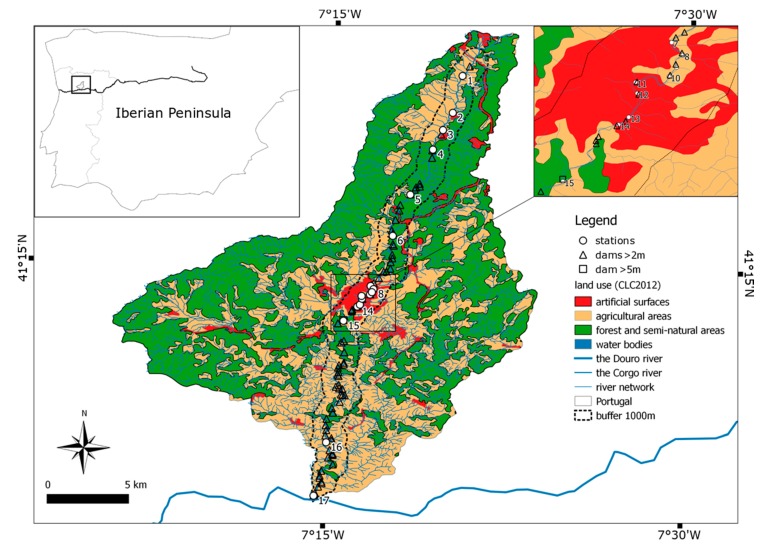
Study area.

This study was conducted in the Corgo river (drainage of the Douro river, Northern Portugal) at 17 sampling sites located in the lotic, free-flowing sections (Figure 1). 

The Corgo catchment is a mountainous basin located in the Trás-os-Montes e Alto Douro province, and the river itself is a tributary in the trans-boundary Douro river basin, within the well-known Douro region—a world heritage site classified by United Nations Educational, Scientific and Cultural Organization UNESCO. The Corgo river rises near the village of Vila Pouca de Aguiar, crosses the town of Vila Real, and reaches its confluence with the Douro river in the village of Peso da Régua. 

**Figure 2:**
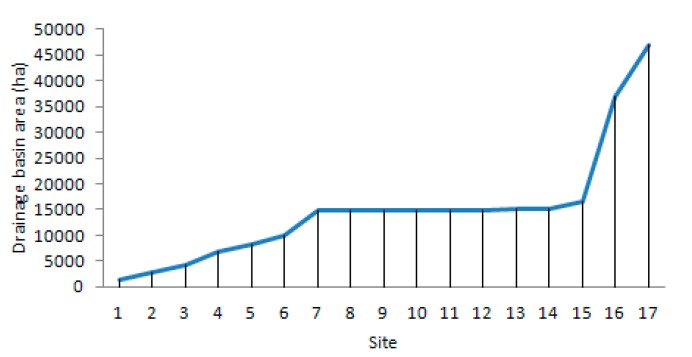
Increase of the drainage basin area of the Corgo river (ha) in the examined sites.

The Corgo river runs to the south, and it rises near the village of Vila Pouca de Aguiar, at an altitude of approximately 918 m. Then, the Corgo meets the Douro river at an altitude of 50 m. The total area of the Corgo catchment is approximately 470 km^2^ (Figure 2). The average riverbed slope is about 2.9%; however, it is not uniform along its course. In general, the main course is steeper when crossing the granites, and becomes slightly smoother towards the mouth, where the schists outcrop. The hillslopes are between 0 and 20% in the upper stretch, and between 20 and 60% in the middle and lower sections.

**Figure 3:**
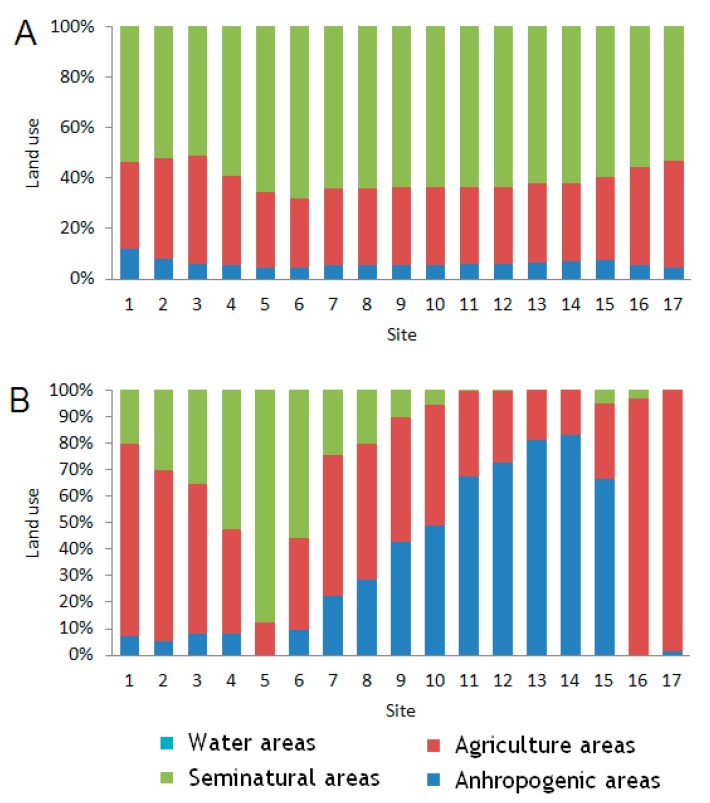
Percentage land use in catchment area of the Corgo river in examined sites. (**A**) Total catchment area. (**B**) Buffer zone of 1 km of catchment area, 2 km upstream.

In terms of land use the Corgo catchment is mostly occupied by forest, natural and seminatural vegetation, and agriculture (Figure 3) (Geographic Information System GIS data). The uncultivated land (36.2%) in the highlands (>650 m) is covered with natural vegetation. Whereas the forest (17%) occurs in limited areas. Agriculture constitutes 42.2% of land use in the basin. The urban land use (4.6%) spreads throughout the basin area; however, it is not uniformly distributed. 

### 2.2. Sampling Methods 

The zooplankton was collected for 3 weeks (weekly) in June of 2015 and 2016 (*n* = 6). At each site, 50 L of water was collected from the river current. The samples were collected using a Van Dorn 5-liter water sampler (KC Denmark, Silkeborg, Denmark) at five depths: 20%, 40%, 60%, 80%, and at the surface [6,14]. At each depth level, 10 L of water was collected to obtain 50 L of water. The water was filtered through a plankton net with a mesh of 30 μm. The samples were then concentrated to 150 mL and fixed in a 4–5% formalin solution. The contents of the samples were counted in a Sedgewick-Rafter counting chamber. A Nikon Eclipse 50i microscope (Nikon, Tokyo, Japan) was used for identification. Afterwards, species were identified using the keys described by Nogrady et al. [18], Janetzky et al. [19], and Błędzki and Rybak [20]. 

As per the Lagrangian scheme, cross-sections were sampled in a downstream sequence with the sampling interval approximating the time of travel between sites. When choosing sampling sites, the authors took the following factors into account: (1) influence of the different catchment uses on the area, (2) influence of the dam impoundments on zooplankton communities downstream, and (3) easy access.

Measurements of temperature, conductivity, and chlorophyll *a* content were made in situ using the Hydrolab DS 5 multiparameter probe (OTT Hydromet, Loveland, CO, USA). At each site, water velocity, width, and depth were measured with an electromagnetic water flow sensor OTT (OTT Hydromet, Loveland, CO, USA) to determine water discharge. A cross-section of the stream channel was divided into five vertical subsections. In each subsection, the area was obtained by measuring the width and depth of the subsection, and water velocity was determined using a current meter. Water discharge in each subsection was calculated by multiplying the subsection area by the measured velocity. The total discharge was then calculated by summing up the discharge values of each subsection. At each site in the 200 m upstream section, we visually estimated the percentage area of the riverbed covered by macrophytes. Table 1 shows values of all measured parameters. All measurements were made on the same day that the zooplankton samples were collected.

The percentage of land use in the catchment was calculated using the Corine Land Cover inventory, 2012 (CLC2012) [21] (European Environmental Agency, European Union). The land use in the catchment was estimated in the buffer zone, which was located within 1000 m of the river shoreline and 2000 m upstream. For our calculations we used the QGIS Wien software (2.8.7) (QGIS Development Team). Based on the findings of Soranno et al. [4], we assumed that the 1000-meter buffer zone located 2000 m upstream has a paramount role in shaping the water chemical components. We took into account all dams with a height of at least 2 m, because smaller dams in the Corgo river caused no impoundments, that could be good basins for zooplankton development. All dams were between 2 and 4 m high except for the dam before Site 15 that was 6 m high. The number of dams is shown in Table 2. 

### 2.3. Data Analyses

Rotifers were divided into two categories according to habitat preference: pelagic species (plankton) and benthic, epiphytic, epilithic species associated with the substratum, otherwise known as benthic species. Copepods were divided into Nauplii, pelagic Cyclopoida (copepodit stages and adults), and Harpacticoida, such as benthic copepods (copepodit stages and adults). All observed Cyclopoida were pelagic species. We used the Kruskal–Wallis test (*p* < 0.05) to check the significance of differences in richness (number of species) and abundance of each zooplankton group between the sites. Post-hoc multiple comparisons of mean ranks for all groups were made (*p* < 0.05) to determine the significant differences in the zooplankton richness and abundance between the sites. To illustrate the ordination of the sites and zooplankton groups in terms of zooplankton group abundance with regard to environmental factors, a canonical correspondence analysis (CCA) was used [22]. The goal of CCA was to determine the similarities between sites and zooplankton groups in terms of the independent variables, in other words, the environmental conditions. The first axis of the CCA represents the strongest variation of zooplankton that we can explain by our environmental variation. The second axis of the CCA is much weaker in explanation of this variation. However, we did not check the significance of the correlations using CCA. In order to determine the significant correlations between environmental factors and the abundance of zooplankton, Spearman’s correlation with Holm’s correction was applied (*p* < 0.05). 

## 3. Results

### 3.1. Taxonomic Composition

Of the 51 taxa of zooplankton observed at all sites, 10 belonged to pelagic Rotifera, 30 to benthic Rotifera, 6 to Cladocera, 3 to Cyclopoida, and 2 to Harpacticoida (Table 3). The highest taxa number was recorded in Site 16, while the lowest number was observed at Sites 6 and 13. Benthic Rotifera definitely reached the highest taxa number at each site. Specifically, the highest number of taxa was recorded at Site 17, and the lowest at Site 2. The taxa number of pelagic Rotifera was small, it ranged from 1 taxon at Site 5 to 6 taxa at Site 7. Similarly, we recorded a small number of taxa of pelagic Rotifera, and it ranged from 1 taxon at Site 5 to 5 taxa at Site 2. Pelagic Copepoda were represented by a low taxa number (2 taxa at Site 3, and 7 was in fact the highest taxa number among this group). Also, there was a small presence of Harpacticoida taxa (max. 2 taxa). 

### 3.2. Species Richness

**Figure 4:**
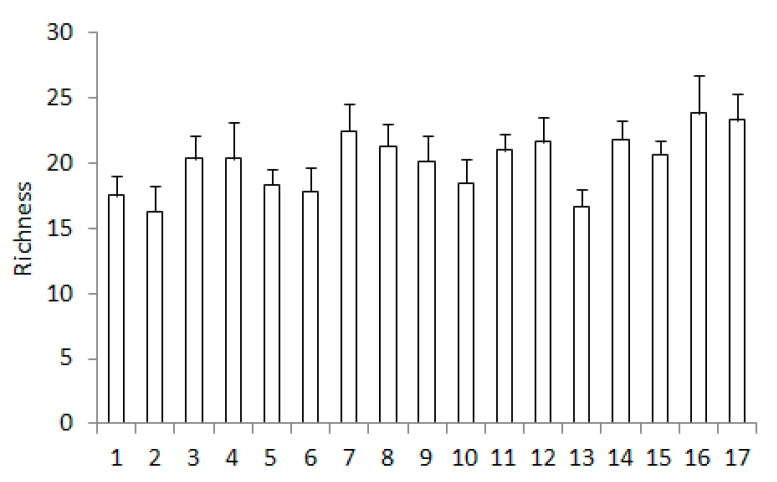
Species richness (mean + SD) of the total zooplankton in the examined sites of the Corgo river.

The lowest mean species richness of zooplankton was recorded at Site 13, while the highest was at Site 16 (Figure 4). Furthermore, we observed a slight upward trend of richness from the sources to the river outlet. Species richness at Sites 16 and 17 was significantly higher than that at Sites 1, 2, and 13 (*p* < 0.05) (Table 4). Also, richness at Site 7 was significantly higher than that at Site 13 (*p* < 0.05).

### 3.3. Abundance

**Figure 5:**
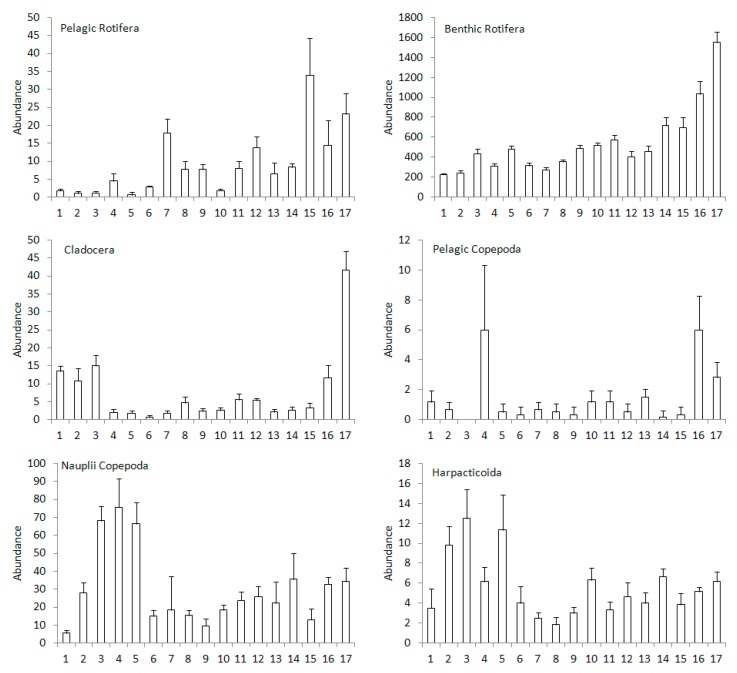
Mean + SD of abundance (ind. 100 L^−1^) of the zooplankton groups in the examined sites of the Corgo river.

In the case of pelagic and benthic Rotifera, an upward trend of abundance from the upper to the lower sections of the river was observed (Figure 5). At Sites 1, 2, 3, 5, 6, and 10 the abundance of pelagic Rotifera was significantly lower than that at Sites 7, 10, 12, 15, 16, and 17 (*p* < 0.05) (Table 5). Abundance of benthic Rotifera at eight sites at the beginning of the river was significantly lower than that at Sites 10, 11, 14, 15, 16, and 17 (*p* < 0.05). The spatial pattern of rotifer abundance was inversely different than that of crustaceans. No upward trend was observed among the crustaceans. The highest abundance of Cladocera was recorded at the first three sites and at the two last sites of the river. Additionally, Cladocera abundance at Sites 1, 2, and 3 was significantly higher than that at Sites 4, 5, 6, and 7 (*p* < 0.05). Moreover, at Site 17 Cladocera abundance was significantly higher than that at the majority of sites (*p* < 0.05), with the exception of Sites 1, 2, 3. Abundance of pelagic Copepoda achieved significantly higher values at Sites 4 and 16 than that at the majority of sites (*p* < 0.05). Abundance of Nauplii Copepods achieved significantly higher values at Sites 3, 4, and 5 than that at Sites 6, 8, 9, and 15 (*p* < 0.05). Whereas the abundance of Harpacticoida was significantly higher at the first sites of the river than that at the majority of sites (*p* < 0.05). In addition, a relatively high abundance of crustaceans was observed at the beginning of the river, while for rotifers this parameter was the highest in the lower section of the river. 

### 3.4. Impact of Environmental Factors

Spearman’s correlation shows that the bigger the catchment area, the greater the abundance of zooplankton (Table 6). Spearman analysis of local relationships between local conditions (in a buffer zone located within 1000 m from the river shoreline and 2000 m upstream) and zooplankton abundance revealed contrasting correlations in terms of pelagic rotifer, and crustacean abundances versus the percentage area of anthropogenic areas. Specifically, pelagic rotifers correlated positively with the percentage of anthropogenic areas, whereas crustaceans correlated negatively with these areas (*p* < 0.05) (Table 7). Agricultural areas correlated positively with cladocerans and pelagic copepod abundance (*p* < 0.05). While semi-natural forest areas correlated negatively with the abundance of rotifers and cladocerans (*p* < 0.05). A positive significant correlation with rotifer abundance and number of dams was also observed, but the number of impoundments close to a site shows the strongest correlation with rotifer abundance (*p* < 0.05). 

Rotifer abundance revealed a significant positive correlation with temperature and discharge (*p* < 0.05) (Table 8). Moreover, a significant positive correlation of rotifer and copepod abundances occurred (*p* < 0.05). Also, the abundance of nearly each zooplankton group significantly increased with higher values of conductivity and chlorophyll *a* content (*p* < 0.05). A negative relationship between pelagic rotifer abundance and macrophyte coverage was observed (*p* < 0.05). 

**Figure 6:**
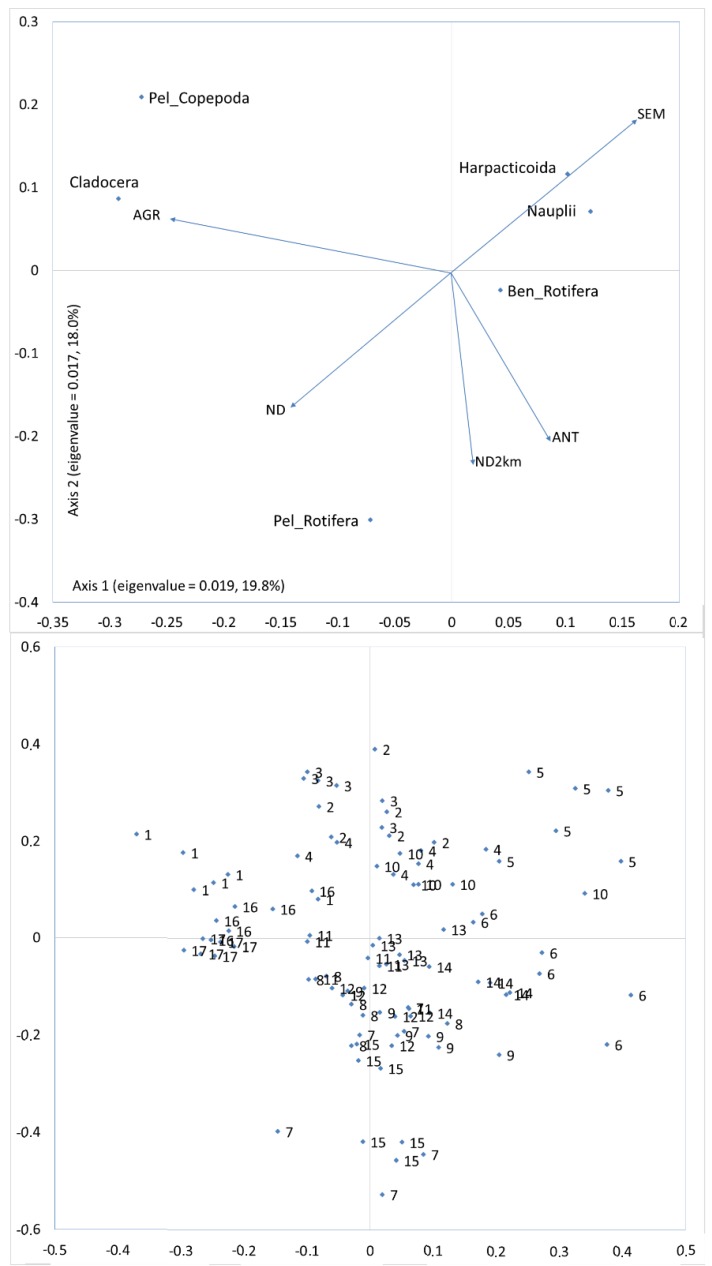
Abundance and factors of the anthropogenic changes in the catchment and in the river bed: canonical correspondence analysis (CCA) constrained ordination of samples and taxa from sites with different current velocity in ditches with the forward selection procedure of environmental variables. Numbers indicate the sites. Environmental variables: AGR—agriculture areas; SEM—seminatural, forest areas, ANT—anthropogenic areas, ND—number of dams above the site in total river length, ND2km—number of dams above 2 km upstream of the examined site.

Two CCA axes explained 37.8% of the total variability in zooplankton group abundance in regard to anthropogenic factors (land use and dam presence) (Figure 6). The agricultural and semi-natural forest areas correlated best with the first axis. The best correlation with the second axis was found for several dams in the 1000-meter buffer zone located 2000 m upstream (ND2km) and the anthropogenic areas. Cladocera and pelagic Copepoda correlated positively with the agricultural areas. Whereas Pelagic Rotifera correlated positively with the presence of anthropogenic areas and dams. These three groups were negatively correlated with the semi-natural forest areas. 

**Figure 7:**
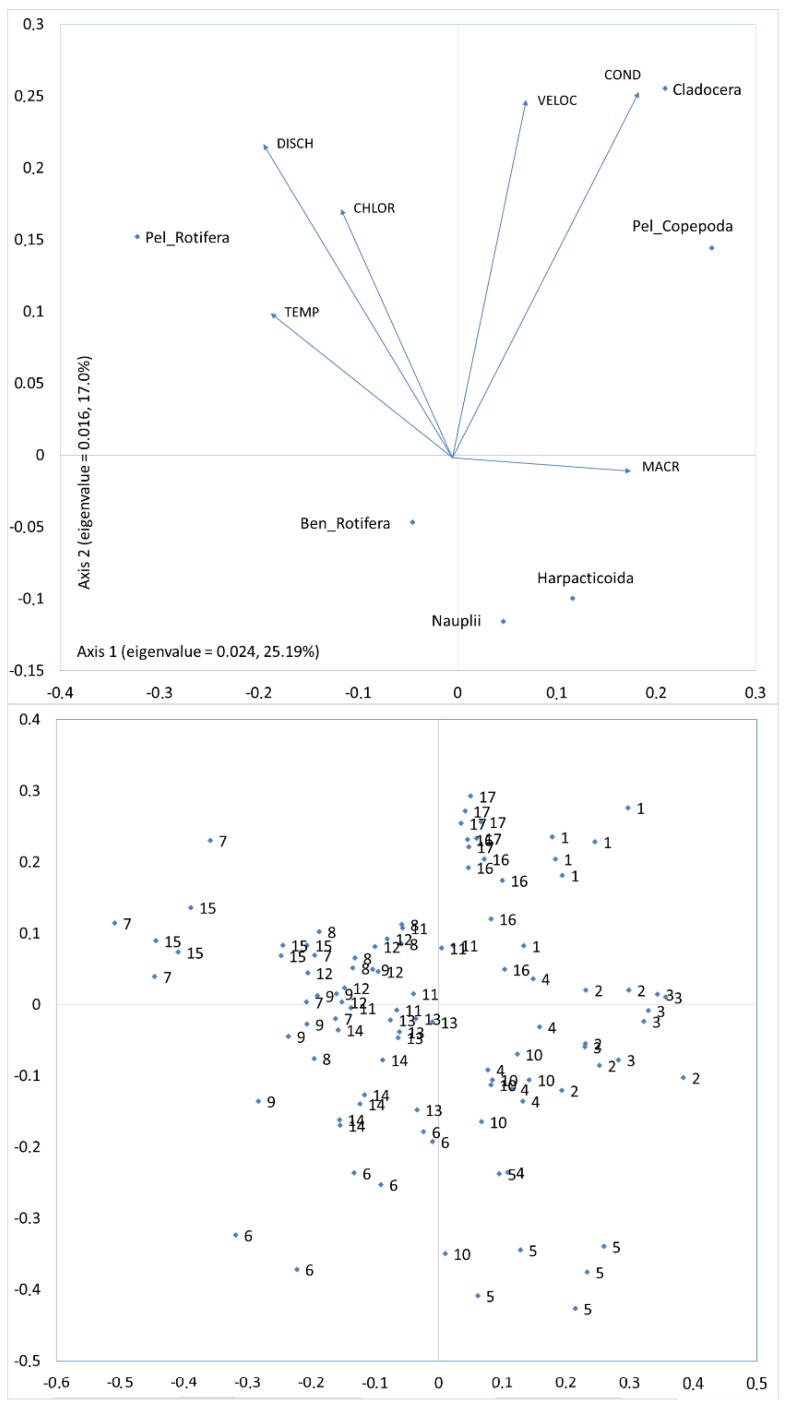
Zooplankton abundance along the environmental factors: CCA constrained ordination of samples and taxa from sites with different current velocity in ditches with the forward selection procedure of environmental variables. Numbers indicate the sites. Environmental variables: VELOC—current velocity; DISCH—discharge; TEMP—temperature; COND—conductivity; CHLOR—chlorophyll *a*.

As for the physicochemical and biological factors, the two CCA axes explained 42.19% of the total variability in zooplankton group abundance (Figure 7). Macrophyte coverage percentage and the discharge, temperature, and conductivity values correlated best with the first axis. Whereas conductivity and current velocity values correlated best with the second axis. Cladocera and pelagic Copepoda correlated positively with the conductivity and macrophytes coverage, while pelagic Rotifera correlated positively with the discharge and temperature values. 

The CCA showed a similar ordination of the sites both in regard to the anthropogenic factors and the physicochemical and biological factors (Figure 6 and Figure 7). Catchment conditions determined a site’s ordination. These conditions affected the values of physicochemical parameters, and in consequence, the zooplankton assemblages. In areas with a large percentage of agriculture land use, the highest values of conductivity were observed. While the sites affected by anthropogenic river bed transformations were characterized by the highest values of chlorophyll a. CCA separated sites with a high influence of agricultural land use (Sites 1, 16, 17) from sites that were most affected by anthropogenic areas with dams (Sites 7, 15) and from sites located in semi-natural areas and forest (Sites 5, 6). Sites in the agricultural areas were the farthest from the sites affected by the semi-natural and forest areas.

## 4. Discussion

Zooplankton communities in large and small rivers are shaped by local conditions of the riverbed, which has been demonstrated in numerous papers [23,24,25,26,27,28]. Due to the greatest abundance of zooplankton being in the downstream section of the Corgo, we could not examine the influence of the entire catchment area on the zooplankton communities because it is evident that the zooplankton density increases with the catchment area size. The above phenomenon is well-known, and it has been defined by the RCC [3]. Therefore, it is more noteworthy to focus on the local conditions [17,29] (such as catchment and river-bed use) and the buffer zone where the influence of the catchment on the nutrient contents [4], and consequently on each zooplankton group, is best seen. Focusing on the entire catchment area seems rather pointless because it is quite unlikely that the upstream environmental conditions would affect the zooplankton communities downstream. Because the rivers have the ability to self-purify, over a few hundred meters there is a significant reduction of plankton observed in lake outflows [13,16].

In the majority of studied rivers there is a typical spatial pattern of various zooplankton groups, and the same was observed in the Corgo. Rotifers were dominant in the lotic waters [23,30]. This was mainly caused by their short development cycle, the ability to rapidly colonize new habitats and to drift passively long distances because of their small specific size and weight [13,16,31,32]. The existence of small reservoirs, even with a relatively short water retention time, determines the large presence of pelagic Rotifera in rivers [9,14,31,33]. Such a pattern was also observed in the Corgo. River-bed transformations, dams, and reservoirs in this small mountain river led to an increase of zooplankton communities in certain sections. The small zooplankton reduction rate was caused by the strong current, which consequently led to a greater zooplankton community downstream than compared to the small lowland rivers [6,14,30]. However, the zooplankton abundance in the Corgo was much smaller than that of small lowland rivers which are subject to anthropogenic changes. The reason for this is the high slope of the Corgo. 

Crustaceans, as opposed to rotifers, occur rarely in small rivers and streams [30,34,35]. Crustaceans have a longer development cycle, and they are heavier and larger in size than rotifers [20]. The above characteristics make it difficult to maintain crustacean populations in rivers and even in small reservoirs. Crustaceans occur in rivers with suitable conditions, such as long water retention time, current velocity up to 0.1 m s^−1^, high open water zones, and the presence of macrophytes [32,36,37,38,39]. In the Corgo, crustaceans were present in sections with such conditions. In its upstream section the Corgo crosses a plateau, and it is at these sites that we recorded the slowest current velocity. Additionally, in that area, the plateau is surrounded by mountains, so there are favorable conditions for agriculture, and as a result there is more nutrient content for the crustaceans in the river. The riverbed in that section was covered mostly by submerged macrophytes. Most importantly, we acknowledge that crustaceans could have migrated to the main channel of the Corgo from the water tanks of a nearby sewage treatment plant and numerous small slackwaters covered by macrophytes [15,27,28,32]. The more extensive was the agriculture use in the catchment area, the greater was the amount of nutrient content, and consequently the more crustaceans were seen. This phenomenon can be confirmed by a high positive correlation between the crustacean abundance and the conductivity. Greater conductivity values occur in waters that are surrounded by agricultural areas [4,40]. 

Crustaceans can be seen in great abundance in small reservoirs, small slackwaters, and macrophyte-covered areas [41,42,43]. Therefore, in the case of pelagic rotifer abundance, we recorded a negative correlation with the percentage of agricultural areas, and a positive correlation with the percentage of anthropogenic areas. The biggest number of impoundments with larger open-water zones was present in the areas subjected to anthropogenic changes. This must have had a positive impact on the abundance of rotifers and crustaceans. However, we believe that the impact was the greatest on the rotifer population. It should be noted that despite the fact that in lotic waters the rotifer abundances are greater than those of crustaceans, similar to the crustaceans, they occur in greater numbers in stagnant waters than in the lotic waters [40]. However, due to short water retention time it was difficult to maintain crustacean population in the Corgo reservoirs. 

In rivers, the only crustacean forms observed in greater amounts are the crustacean larvae. Such a pattern was also observed in the Corgo. At each site, Nauplius occurred in greater numbers than their adult forms. Oftentimes in rivers, larvae abundance is greater than that of adult crustaceans [30,32,44]. Similar to the rotifers, and due to the same reasons, nauplii as small plankters drift much farther than adult crustaceans.

As mentioned above, more extensive agriculture in a catchment area is the factor which confirms the positive influence of anthropic activity on the zooplankton abundance, and especially the rotifers. In the present study, this factor was expressed with a greater number of dams and reservoirs in the vicinity of agricultural and urban settlements, which had an impact on relatively large open-water zones that were much larger than those in the upper section of the Corgo. The presence of impoundments was highly positively correlated with the zooplankton abundance, and specifically small rotifers [13,15,31]. Not only do the impoundments prolong the water retention time, but they increase the river temperature and the amount of nutrient content. Furthermore, this phenomenon was confirmed by the positive correlation between the zooplankton abundance and water temperature and the chlorophyll *a* values observed in this study. This relationship is widely known because zooplankton are primarily dependent on primary producers and on bacteria, flagellates, and ciliates [17,45,46,47].

No positive correlations between zooplankton abundance and forest areas were found, because in these sections there are no dams or natural water reservoirs where zooplankton could develop and then disperse into the Corgo sections along the forest areas, where the natural bed was not affected by human activity. 

As mentioned in the Introduction, it is highly unlikely that anthropogenic changes in the upper section of the Corgo affected the zooplankton communities in the middle and lower sections in any way. The species that dominated upstream were very scarce in the lower sections of the river. Therefore, the influence of river environmental conditions on the zooplankton occurs locally. The abundance of zooplankton, and more specifically the rotifers, may increase due to river hydrological conditions and the presence of impoundments. Certain authors believe that zooplankton can drift over long distances due to high current velocities and turbulence, which prevent sedimentation phenomena and also prevent fish and macroinvertebrates from feeding on plankters [13,16,24,48,49]. This is true in the case of the Corgo, and it was confirmed by a positive correlation between current velocity and rotifer abundance, which is not likely to be found in lowland rivers [34,35,39]. Contrary to mountain rivers, lowland rivers are characterized by lower current velocities and weaker turbulences, which in turn may lead to greater reduction in plankton abundance. However, both in lowland and mountain rivers such a phenomenon is shaped by local conditions.

## 5. Conclusions

It seems that contrary to small lowland rivers, water retention time in reservoirs in small mountain rivers is so short that only small pelagic rotifers, characterized by a short and fast reproductive cycle, can maintain their population. Crustaceans require much longer water retention time. Therefore, crustaceans present in the Corgo can attain relatively high densities in the rural section, which offers them better trophic conditions. Additionally, in this section, current velocity is low, and slackwaters (from which crustaceans are washed out) are connected with river beds and sections densely covered by submerged macrophytes. The dams on the Corgo unequivocally increase the water retention time; however, this period is too short for crustaceans to maintain their populations, but it is sufficient for the rotifers. Therefore, in the Corgo, the urban catchment-use and the presence of dams have a greater impact on the rotifer density and the increase of zooplankton density downstream. Whereas the agricultural catchment-use as local conditions affect the crustacean density, and to a lesser extent the rotifer density. Semi-natural forest catchment had no effect on the increase of zooplankton abundance. Moreover, a reduction in plankton abundance drifting from upstream, that is typical of rivers, occurred in the forest section. Our results show that impounded sections of the Corgo river were characterized by more numerous zooplankton communities than were the unimpounded sections. However, this pattern was observed within a confined geographical area within the same drainage.

In closing, it is worth noting that zooplankton richness and abundance can be a good indicator of trophic status [40,50,51,52,53], river-bed transformations, and the use of the riparian zone or the catchment area. Alas, this factor has not been included among the Water Framework Directive’s biological indicators used to assess freshwaters. However, a few years ago, zooplankton were proposed as a good bioindicator of lake conditions [50,51,52]. Moreover, the results of this study as well as the research of others confirm that zooplankton properties allow for the evaluation of the degree of river-bed transformations.

## Figures and Tables

**Table 1:** Mean values of environmental factors measured at examined sites of the Corgo river.

Site	Temperature	Conductivity	Chlorophyll *a*	Macrophyte Coverage	Current Velocity	Discharge
	(°C)	(µS·cm^−1^)	(µg·L^−1^)	(%)	(m·s^−1^)	(m^3^·s^−1^)
1	13.7	83	0.02	20	0.45	0.394
2	15.0	84	0.07	5	0.50	0.408
3	14.4	78	0.35	73	0.46	0.454
4	16.4	57	0.50	2	0.05	1.087
5	16.7	54	0.32	0	0.05	1.322
6	16.0	47	0.18	0	0.05	1.491
7	15.8	47	0.12	0	0.57	3.602
8	16.6	50	0.05	0	0.39	3.846
9	17.4	51	0.15	1	0.30	4.140
10	16.6	53	0.27	5	0.25	4.369
11	17.3	57	0.95	3	0.20	4.780
12	17.9	53	0.88	1	0.25	5.079
13	20.2	50	0.93	11	0.07	5.823
14	19.6	62	0.77	22	0.56	5.846
15	18.9	73	1.60	16	0.05	7.775
16	20.0	105	1.42	0	0.59	8.897
17	20.3	113	1.22	1	1.17	10.389

**Table 2:** Number of dams in the Corgo river above the examined site (ND) and more than 2 km upstream of the examined site (ND2km).

Site No.	1	2	3	4	5	6	7	8	9	10	11	12	13	14	15	16	17
ND	1	1	2	4	10	16	27	28	29	29	30	31	32	33	38	64	78
ND2km	1	0	1	2	5	4	5	4	5	5	6	6	5	5	5	4	7

**Table 3:** Taxonomic composition and percentage contribution of zooplankton taxa in mean abundance of total zooplankton at examined site.

Taxa	1	2	3	4	5	6	7	8	9	10	11	12	13	14	15	16	17
Pelagic Rotifera																	
*Ascomorpha ovalis*															<	<	<
*Brachionus calyciflorus*	<	<		<	<												
*Kellicottia longispina*							<		<	<	<	<					
*Keratella cochlearis*	<	<	<	<	<	<	2	1	1	<	<	<	<	<	<	<	<
*Keratella quadrata*							1	<	<								
*Notholca foliacea*							<	<									
*Polyarthra remata*																<	<
*Trichocerca intermedia*	<			1	<	1	2	1	1	<	1	3	1	1	4	1	1
*Trichocerca tenuior*							<										
*Trichocerca* sp.															<	<	<
Benthic Rotifera																	
*Cephalodella auriculata*				<													
*Cephalodella forficula*				<	<	<	1										
*Cephalodella gibba*	1			<	<	1	<	<	<	<	1	<	<	<	2	1	1
*Cephalodella gracilis*							<										
*Cephalodella sterea*															<		
*Cephalodella ventripes*	<	<	<	<	<	1	<	<			<	1	<	<	<	<	<
*Cephalodella sp.*	1	<	<	3					1	<	1	<	<	4	1	2	2
*Colurella adriatica*	28	9	13	16	13	22	17	30	25	18	28	24	40	28	23	15	9
*Colurella colurus*	2	6	<	<	1	1		<	1	1	<	<	<	1	2	<	<
*Colurella uncinata*	<	<	<	<		<	<	<			<			2		<	<
*Euchlanis dilatata*	1	<	1	1	1		<	1	2	3	<	8	17	4	2	2	1
*Euchlanis lyra*							<						<				
*Lecane arcuata*								1	<	<	<	1		<		<	<
*Lecane bulla*	11	10	5	10	11	14	15	16	11	14	11	14	9	8	6	6	9
*Lecane closterocerca*	5	8	10	5	14	9	6	3	11	7	6	7	4	6	6	6	9
*Lecane flexilis*	1			<	<		1			<	<	1	<	<	1	1	2
*Lecane hamata*	<	<	1		1				3	3	2	<		1		<	<
*Lecane levistyla*														<			
*Lecane ligona*		<		<	<	1		<	<			<		<			<
*Lecane lunaris*	1	5	7	7	12	7	7	8	3	13	13	5	7	10	5	9	4
*Lecane mira*			<		3	2	4	4	3	1	10	4	1	9	2	2	<
*Lecane tenuiseta*			<					<							2		
*Lecane* sp.	2	9	5	<	5	1	3	1	5	6	5	5	2	5	<	4	1
*Lepadella acuminata*	1	2	1	1	2	2	3	2	1	<	2	1	<	<	3	<	<
*Lepadella elliptica*			<		<			<			<	<	<	<		<	
*Lepadella ovalis*	10	20	12	9	17	8	6	6	4	8	4	4	3	6	6	3	3
*Lepadella rhomboides*						1											
*Monommata actices*																	<
*Ploesoma triacanthum*																	<
Bdelloidea	28	22	37	37	18	28	30	24	26	23	12	19	12	13	32	43	51
Cladocera																	
*Daphnia longispina*						<											
*Bosmina longirostris*	5	3	1	<	<		<	<	<	<						<	<
*Alonella nana*		1	1	<			<	<	<	<	<	<					
*Chydorus sphaericus*	<	<	<					<									
*Alona costata*		<	1	<		<	<	1	<	<	1	1	<	<	<	1	2
*Alona guttata*	<	<															
Pelagic Copepoda																	
*Acanthocyclops trajani*	<		<														
*Mesocyclops leuckarti*							<										
*Eucyclops serrulatus*			<	<			<										
Copepodit Cyclopoida	<	<	<	1	<	<		<	<	<	<	<	<			1	<
Copepodit Calanoida														<	<		
Nauplii Copepoda																	
Nauplii Copepoda	<	<	<	2	<	<	<	<	<	<	<	<	<	<	<	1	<
Harpacticoida																	
*Bryocamptus minutus*	1	1	1	1	1	<	<	<	<	<	<	1	<	<	<	<	<
*Bryocamptus pygmaeus*				<		<	<			<			<	<			<
Copepodit Harpacticoida	1	3	1	1	1	1	<	<	<	1	<	<	<	1	<	<	<

< identifies taxa with a contribution lower than 1.

**Table 4:** Significant differences (*p*–values) in total zooplankton species richness between examined sites. Numbers indicate site name, * *p* < 0.05, ** *p* < 0.01.

Site	1	2	7	13
7		*		
13			*	
16	*	**		**
17	*	**		**

**Table 5:** Significant differences (*p*–values) in zooplankton abundance between the examined sites. Numbers indicate site name, * *p* < 0.05, ** *p* < 0.01, *** *p* < 0.01.

**Pelagic Rotifera**	**Site**	**1**	**2**	**3**	**5**	**6**	**7**	**10**				
	7	*	**	**	**							
	10						*					
	12		*	*	**							
	15	**	***	***	***	*		**				
	16		*	*	*							
	17	**	***	***	***			**				
**Benthic Rotifera**	**Site**	**1**	**2**	**4**	**6**	**7**	**8**					
	10	*										
	11	**	*									
	14	***	**			**						
	15	***	**			*						
	16	***	***	**	**	***						
	17	***	***	**	**	***	*					
**Cladocera**	**Site**	**1**	**2**	**3**	**4**	**5**	**6**	**7**	**9**	**10**	**13**	**14**
	4			*								
	5	*		*								
	6	***	**	***								
	7	*		*								
	16						**					
	17				**	**	***	**	*	*	**	*
**Pelagic Copepoda**	**Site**	**4**	**5**	**6**	**8**	**9**	**12**	**14**	**15**			
	6	*										
	9	*										
	14	**										
	15	*										
	16		*	*	*	*	*	**	*			
	17							*				
**Nauplii Copepoda**	**Site**	**1**	**3**	**4**	**5**							
	3	***										
	4	***										
	5	***										
	6		*	*	*							
	8		*	*	*							
	9		***	***	**							
	15		***	**	*							
	17	*										
**Harpacticoida**	**Site**	**1**	**2**	**3**	**5**	**8**						
	3	*										
	5	*										
	7		**	***	**							
	8		***	***	***							
	9		**	**	**							
	11		*	**	*							
	14					*						
	15			*								

**Table 6:** Spearman significant correlations with Holm’s correction between zooplankton abundance (ind. l^−1^) and percentage land use in the total catchment area (ha).

Land Use	Pelagic Rotifera	Benthic Rotifera	Cladocera	Pelagic Copepoda
Anhropopressure	0.75	0.83		
Agriculture	0.74	0.85		
Seminatural	0.74	0.80		
Water basins			0.50	0.25

**Table 7:** Spearman significant correlations with Holm’s correction between zooplankton abundance and percentage land use in the local environment of the sites in the Corgo river (1 km buffer zone 2 km upstream) and between the number of dams above the site in the total river length and above 2 km upstream of the examined site (ND2km) (*p* < 0.05).

Land use/Dams	Pelagic Rotifera	Benthic Rotifera	Cladocera	Pelagic Copepoda	Naupli Copepoda	Harpacticoida
Anthropopressure	0.30		−0.33	−0.37	−0.31	−0.34
Agriculture			0.62	0.42		
Seminatural	−0.58	−0.60	−0.25			
ND	0.58	0.59				
ND2km	0.76	0.83				

**Table 8:** Spearman significant correlations with Holm’s correction between zooplankton abundance and environmental variables in the sites of the Corgo river (*p* < 0.05).

Environmental variables	Pelagic Rotifera	Benthic Rotifera	Cladocera	Pelagic Copepoda	Naupli Copepoda	Harpacticoida
Temperature	0.62	0.77				
Conductivity		0.35	0.78	0.44	0.30	0.41
Chlorophyll *a*	0.54	0.78		0.28	0.30	
Macrophyte coverage	−0.24					
Current velocity	0.25		0.59			
Discharge	0.76	0.82				
